# Four different malignancies in one patient: a case report

**DOI:** 10.1186/1757-1626-3-53

**Published:** 2010-02-08

**Authors:** Umut Demirci, Ugur Coşkun, Pinar Uyar Göçün, Bahar Gurlek, Burcu Saka, Banu Öztürk, Mustafa Benekli, Süleyman Büyükberber

**Affiliations:** 1 Department of Medical Oncology, Faculty of Medicine, Gazi University, Ankara, Turkey; 2 Department of Pathology Faculty of Medicine, Gazi University, Ankara, Turkey; 3 Department of Internal Medicine, Faculty of Medicine, Gazi University, Ankara, Turkey

## Abstract

Cancer survivors have a higher risk of new primary cancer, in the same or in another organ, than the general population. We report a 78-year-old women who has metachronous quadruple adenocarcinoma, includes bilateral breast cancer, ovarian cancer and retroperitoneal neuroendocrine carcinoma. The development of second cancer in cancer survivors can be expected but third or higher order malignancies are rare.

## Introduction

Cancer survivors are a growing group owing to improvements in widely scanning and treatment. In this group the most serious event is the diagnosis of a new second cancer. Also older people population increases. Thus two reasons occurrence of multiple primary cancers are likely to increase.

Cancer patients have a 20% higher risk of new primary cancer, in the same organ or in another organ, compared with the general population. Also, second cancers have become a leading cause of death among long-term cancer survivors [1]. Individual susceptibility factors remain largely unknown but, it may not necessarily be attributable solely to prior cancer treatment but may also reflect the effect of shared etiologic factors, environmental exposures and inherited predisposition. Multiple cancers, two or more primary cancers, were defined: each of the tumors must present a definite pattern of malignant disease, each must be distinct, and the possibility that one tumor is a metastasis of the other must be excluded since 1932 [2].

In cancer survivors, the number of second- or higher-order cancers is burgeoning and accounted for about 16% of incident cancers in 2003 [3]. The development of second cancer in cancer survivor is expected but third, or higher order malignancies are rare. To our knowledge, this is the first detected case with this combination of primary adenocarcinomas.

## Case

**Figure 1:**
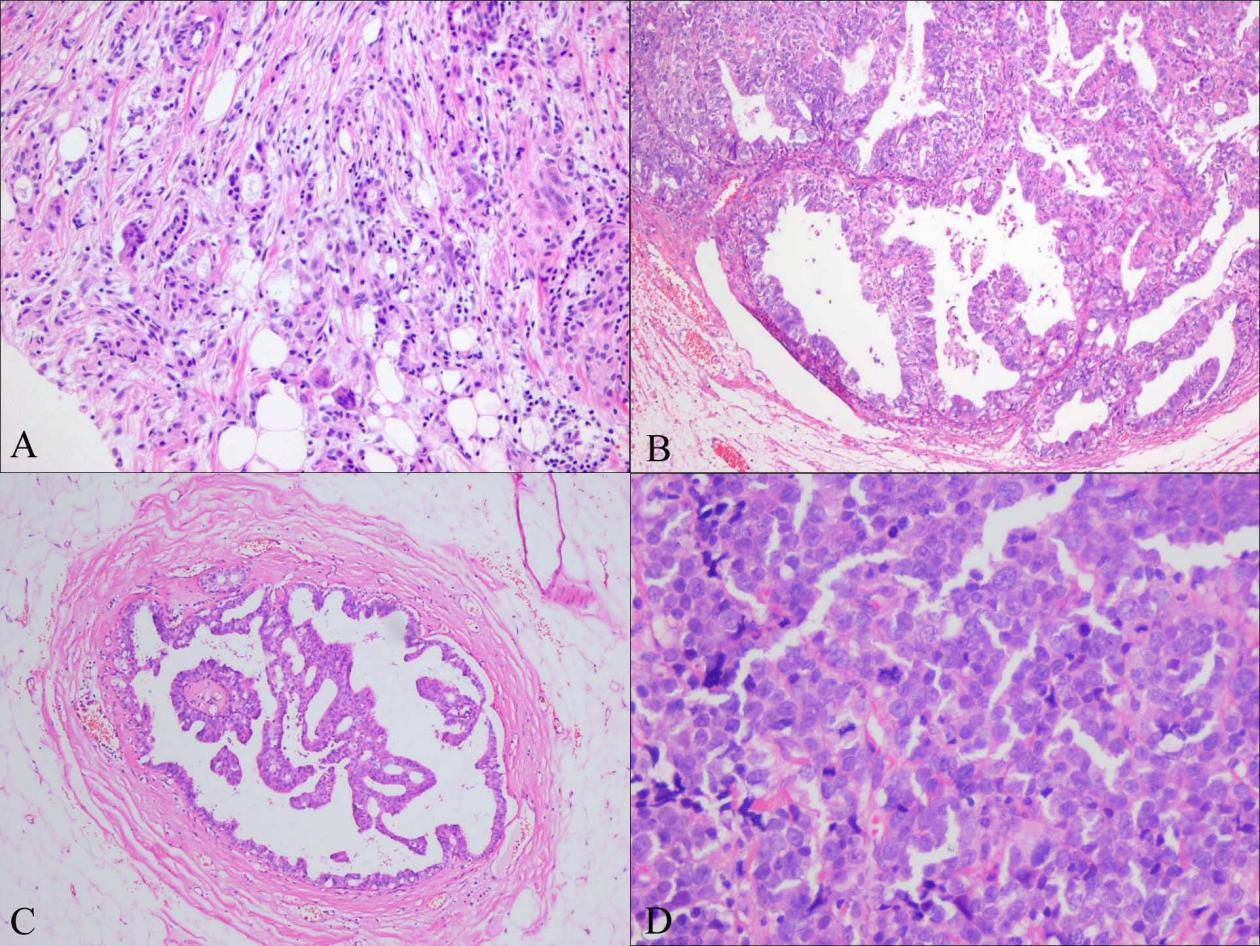
**A-Invazive ductal carcinoma; Glandular differentiation is apparent as tubular structures with central lumina**. A proportion of tumour cells are arranged in cords, clusters and trabeculae (HE ×10), B- Poorly differentiated serous carcinoma glandular and papillary architecture of tumour. The glands are typically irregular and slit-like (HE ×10), C- Intraductal carcinoma with cribriform and micropapillary pattern (HE ×40), D- Undifferantiate neuroendocrine carcinoma; sheets of polygonal, round cells with salt and pepper nuclei and numerous mitotic figures (HE ×10).

A 78-year-old patient who had metachronous quadruple cancer was admitted to our clinic. In her family history; her grandmother had lung cancer. She had undergone right modified radical mastectomy for invasive breast cancer (Fig. 1-A) in 1996. After adjuvant chemotherapy and radiotherapy she treated with hormone therapy as tamoxifen. After two years, in 1998, she had undergone total abdominal hysterectomy, bilateral salphingoopherectomy (TAH&BSO) and partial omentectomy for her right ovarian mass. Pathologic examination showed poorly differentiated serous carcinoma (Fig. 1-B). No metastasic lesion was detected. She treated with combined paclitaxel and cisplatin regimen for 6 cycles. Then, she was followed-up without any sign of recurrence. She felt accidentally a lump of the left breast so she had visited a hospital for a work-up in 2006. The detailed examination proved that the intraductal carcinoma (Fig. 1-C) in left breast mass was primary breast cancer so the right modified radical mastectomy was carried out and pathologic examination showed intraductal carcinoma. Hormone receptor status was negative. She did not have any adjuvant treatment. Lobular border solid mass in left renal hilus was detected by abdominal computerized tomography (CT). Trucut biopsy was done and pathologic examination showed undifferantiate neuroendocrine carcinoma (Fig. 1-D). In the surgery abdominal aorta was surrounded by tumor in pancreas tail. It was accepted as in-operabl. She was treated with combined cisplatin and etoposid chemotherapy regimen. After 3 cycles response was evaluated as partial response and stable disease after 5 cycles of chemotherapy. In August 2008, her clinical status was deterioted and detected as progressive disease and combination carboplatin and etoposide regimen was administered.

## Discussion

We report an unusual case of metachronous quadruple which were all adenocarcinomas. She had undergone surgery and combined chemotherapy for these cancers. Multiple primary malignancies are common, in a study encountered in 3-5% of malignant tumors which are most often secondary, triple tumors occur in only 0.5%, quadruple tumors in 0.3% of malignant tumors [4]. Our patient has a quadruple cancer accordingly Agency for Research on Cancer (IARC) but not according to Surveillance Epidemiology and End Results (SEER) rules. Contralateral malignant lesions of the breast are considered as subsequent primary tumours according to SEER rules [5].

The increased risk of subsequent malignancies among cancer survivors is well-established and initially diagnosed with cancer ages 30 to 49. Second primary cancers can be examined into three categories; therapy releated, syndromic and those resulting from shared etiologic influences by Travis et al [6]. SEER program data were diagnosed with a second cancer by 25 years follow-up for patients who have already developed a second malignancy, recognition of the types of possible additional malignancies, the associated latency periods, and underlying risk factors such as treatment, genetic predisposition, environmental cofactors will have important implications for follow-up and screening. In general second malignancies are a result of success and are not observed unless a patient survives an initial cancer diagnosis [3].

Deligdisch et al. analyzed that 67 (5.4%) cases of multiple primary malignancies of the genital organs and breast in their different combinations in 1235 multiple primary malignancies. A higher incidence of multiple primary cancers were found in embryologically related organs such us endometrium and ovary [7]. Similar results were reported as the most frequent organ involved in multiple tumors was breast, the largest amount of data exist for contralateral breast cancer, probably due to embriologic or common etiologic factors. Like in our patient association breast and ovarian cancers are likely due to genetic factors and hormonal influences. Cancer treatment (chemotherapy and radiotherapy) and insufficient hormone therapy may cause contralateral breast cancer in our case. However familial cancer syndrome should be thought because of combinations of ovarian and breast cancer, although our case had no evident family history of cancer.

Watanabe et al. analyzed multiple primary malignancies in 285 (5.2%) double primary cancers, 58 (1.1%) triple or more in 5,456 consecutive autopsy cases [8]. In Antal et al.'s study with 719 cancer patients, multiple malignancies were found in 53 cases (7.4%). 49 of these being second malignancy and 4 were third malignancy. Colorectal and gynecological malignancies appeared with breast cancer in 5 cases [9]. Ng et al. reported 181 patients second malignancies in 1,319 Hodgkin lymphoma patients. From these, 18 ot them developed a third malignancy, especially breast cancers, lung cancers and genitourinary cancers. The median time between the development of the second and third malignancy was 34 months [10]. Bhatia et al. described 141 patients developed 1 subsequent malignancy, 26 patients developed 2 subsequent malignancies, 5 patients developed 3 subsequent malignancies, and one patient developed 4 subsequent malignancies among 1,380 childhood cancer survivors. The third neoplasms included 28 solid malignancies, 1 hematologic malignancy. The estimated 10-year cumulative incidence of developing a third malignancy was 21% from the time of diagnosis of the second malignancy [11]. Latent period between second and third malignancies was 8 years. Our case was not similar to data in the literature for this reason.

In our patient, Neuroendocrine tumors (NET) was the forth developed cancer and they are frequently associated with synchronous or metachronous secondary malignancies. Progemmer et al. showed that 14 patients with NET and secondary malignancies from a series of 96 patients with NET. The median age of the patients at diagnosis of NET was 69 years (range: 56-86 yrs). 5 patients had synchronous secondary malignancies and 9 metachronous secondary malignancies [12].

Our case has metachronous quadruple adenocarcinomas and she may develop fifth or more malignancy in the following times. In conclusion, we need an improvement for our knowledge of the risks and patterns of high-order malignancies.

## Consent

Written informed consent was obtained from the patient for publication of this case report and any images. A copy of the written consent is available for review by the editor-in-Chief of this journal.

## Competing interests

The authors declare that they have no competing interests.

## Authors' contributions

UD conceived the study. PUG, BG, BS, BO, MB and SB performed the literature review. UD, and UC edit and coordinated the manuscript. All authors read and approved the final manuscript.
